# Delivery outcomes and patterns of morbidity and mortality for neonatal admissions in five Kenyan hospitals

**DOI:** 10.1093/tropej/fmv024

**Published:** 2015-04-04

**Authors:** Jalemba Aluvaala, Dorothy Okello, Gatwiri Murithi, Leah Wafula, Lordin Wanjala, Newton Isika, Aggrey Wasunna, Fred Were, Rachael Nyamai, Mike English

**Affiliations:** ^1^KEMRI-Wellcome Trust Research Programme, Nairobi, Kenya; ^2^Department of Paediatrics and Child Health, University of Nairobi, Nairobi, Kenya; ^3^Kenya Paediatric Association; ^4^Ministry of Health, Government of Kenya; ^5^Nuffield Department of Medicine, University of Oxford, Oxford, UK; ^6^Department of Paediatrics, University of Oxford, Oxford, UK

**Keywords:** Neonatal morbidity and mortality, maternal mortality, still births, hospital care, developing countries

## Abstract

A cross-sectional survey was conducted in neonatal and maternity units of five Kenyan district public hospitals. Data for 1 year were obtained: 3999 maternal and 1836 neonatal records plus tallies of maternal deaths, deliveries and stillbirths. There were 40 maternal deaths [maternal mortality ratio: 276 per 100 000 live births, 95% confidence interval (CI): 197–376]. Fresh stillbirths ranged from 11 to 43 per 1000 births. A fifth (19%, 263 of 1384, 95% CI: 11–30%) of the admitted neonates died. Compared with normal birth weight, odds of death were significantly higher in all of the low birth weight (LBW, <2500 g) categories, with the highest odds for the extremely LBW (<1000 g) category (odds ratio: 59, 95% CI: 21–158, *p* < 0.01). The observed maternal mortality, stillbirths and neonatal mortality call for implementation of the continuum of care approach to intervention delivery with particular emphasis on LBW babies.

## INTRODUCTION

Global reports indicate that the highest risk of neonatal death is in Sub-Saharan Africa, with Kenya among the 10 countries contributing most deaths [1]. With high coverage of basic interventions [2], up to 71% of neonatal deaths could be averted with >82% of this effect attributable to facility-based care [3]. However, national, and thus global, reports are based on limited data on neonatal case-mix and outcomes; the available data are largely derived from episodic, limited-scale surveys [4]. Further, the national hospital information management system (HMIS) in Kenya has been shown to have poor-quality data [5]. There are particularly few data exploring possible variability in neonatal case-mix and outcomes. This study, therefore, sought to use data (collected specifically for the study separate from the national HMIS) from five Kenyan hospitals, from a continuum of care perspective [6, 7], to (i) profile maternal characteristics, (ii) determine delivery outcomes (still births and maternal mortality), (iii) document the causes of neonatal admissions and (iv) examine the effect of birth weight on neonatal mortality.

## METHODS

This was a cross-sectional survey conducted in neonatal and maternity units of five Kenyan urban public hospitals in November and December 2013. Data were abstracted retrospectively from admission registers covering a 1 year period (October 2012–September 2013). These included data from all inborn neonatal admissions plus maternal data abstracted from a sample of 800 deliveries per hospital. In addition, for stillbirths, live births and maternal mortality, a tally of the total number of events over the 1 year period was obtained. The data were entered directly into REDCap® electronic data capture tools. Data quality was checked in real time by checks built into REDCap. In addition, at the end of each day, these data would be transmitted to a central server in KEMRI-Wellcome Trust where a STATA version 12 (Stata Corporation, Texas, USA) check file was run and a list of potential errors generated and sent back to the sites for verification and correction. Analyses were also done in STATA version 12. Pooled results are presented with 95% confidence intervals (CI), while the association between birth weight and neonatal mortality was examined using a random effects logistic regression model. The effect of clustering at hospital level was taken into account in these analyses.

## RESULTS

A total of 3999 maternal records were sampled but variation in missingness was observed across the maternal characteristics resulting in different denominators. Teenage (13–19 years) mothers accounted for 19% (745 of 3938, 95% CI: 12–26%) of these records. Primi gravidae mothers constituted 42% (1661 of 3959, 95% CI: 38–45%) and grand-multiparous (≥5 live births) were 2.4% (96 of 3959, 95%CI: 0.4–5%) of the sample. Overall, <10% of the mothers were human immunodeficiency virus positive (7%, 230 of 3462, 95% CI: 3–19%) but within hospitals this ranged from 2 to 16% (13 of 659, 95% CI: 1–3%, and 120 of 736, 95% CI: 14–19%, respectively). By contrast, syphilis, tested by Venereal Disease Research Laboratory (VDRL) test, was positive in 1% (29 of 3467, 95% CI: 0.3–1.2).

Extremely low birth weight (LBW) babies (<1000 g), very LBW babies (1000 to <1500 g) and all LBW babies combined (<2500 g) constituted 0.4% (14 of 3826, 95% CI: 0.02–0.7%), 1.3% (48 of 3826, 95% CI: 0.7–1.7%) and 10% (394 of 3836, 95% CI 7–14%) of all sampled deliveries, respectively. Gestation at delivery was poorly documented (51% missing) and therefore not reported. There were 37 still births per 1000 births (559 of 15 050, 95% CI: 34–40 per 1000), but with variation across hospitals (range 11–43 per 1000). Forty maternal deaths [maternal mortality ratio (MMR) 276 of 100 000 live births; 95% CI: 197–376] were recorded; the highest number of maternal deaths per facility was 14, lowest 4.

A total of 1836 inborn admissions to the neonatal units were documented (Table 1). These admissions comprised 13% of live births (1836 of 14 491, 95% CI: 12–13%). Gestation at delivery was universally missing from the neonatal unit admission registers. Most admissions were on the first day of life (72%; 1246 of 1736, 95% CI: 16–97%). Diagnoses are presented as disease episodes, meaning patients with multiple diagnoses contributed a count in each diagnosis. The top three diagnoses at admission were birth asphyxia (30%), prematurity/LBW (28%) and neonatal sepsis (14%). A fifth (263 of 1384, 19%, 95% CI: 11–30%) of the neonatal admissions died (Table 2). Extremely LBW and very LBW accounted for 3% (43 of 1576, 95% CI: 2–4%) and 9% (144 of 1576, 95% CI: 5–16%) of admissions with case fatality of 84% (32 of 38, 95% CI: 45–97) and 61% (77 of 126, 95% CI: 42–77%), respectively. All LBW accounted for 38% (604 of 1576, 95% CI: 28–50%) of admissions and 68% of deaths (179 of 263, 95% CI: 63–73%).

**Table 1.** Patient characteristics and patterns of morbidity in neonatal admissions

Characteristic	H1 *n*^a^^ ^= 211		H2 *n*^a^^ ^= 693		H3 *n*^a^^ ^= 235		H4 *n*^a^^ ^= 400		H5 *n*^a^^ ^= 297		Total^b^ *n*^a^^ ^= 1836	
	*n*^c^	Est.^d^	*n*^c^	Est.^d^	*n*^c^	Est.^d^	*n*^c^	Est.^d^	*n*^c^	Est.^d^	*n*^c^	Est.^d^
Age days (med, IQR)	208	0 (0–1)	693	0 (0–0)	198	1 (1–1)	341	1 (0–2)	296	0 (0–0)	1736	0 (0–1)
Sex (%, 95% CI)												
Female	87	43 (36–50)	331	49 (45–53)	94	44 (38–51)	125	37 (32–43)	111	38 (32–43)	748	43 (36–52)
Total	202		677		212		335		294		1,720	
Birth weight^e^ (%, 95% CI)												
Extremely LBW	3	1.5 (0.3–4)	18	2.7 (2–4)	2	1.4 (0.2–5)	7	2.6 (0.7–5)	13	4.4 (2–7)	43	3 (2–4)
Very LBW	34	17 (12–22)	41	6.2 (4–8)	9	6.3 (2–10)	34	13 (9–17)	26	8.8 (6–12)	144	9 (5–16)
LBW	65	32 (26–38)	151	23 (20–26)	29	20 (14–27)	77	29 (23–34)	95	32 (27–37)	417	27 (20–34)
Normal	93	46 (39–53)	423	64 (60–67)	91	64 (56–72)	137	51 (45–57)	157	53 (47–59)	901	57 (46–67)
Macrosomia	8	4 (1–7)	32	5 (3–6)	11	8 (3–12)	14	5 (3–8)	6	2 (0.4–4)	71	5 (3–7)
Total	203		665		142		269		297		1,576	
Admission diagnoses^f^ (%, 95% CI)												
Birth asphyxia	90	43 (35–49)	177	26 (22–29)	81	35 (28–41)	106	27 (22–31)	105	35 (30–41)	559	31 (23–39)
Premature/LBW	94	45 (38–51)	158	23 (20–26)	69	29 (25–35)	114	29 (24–33)	91	31 (25–36)	526	29 (21–38)
Neonatal sepsis	15	7 (4–11)	90	13 (11–16)	52	22 (17–28)	88	22 (18–26)	14	5 (2.3–7.1)	259	14 (8–25)
Newborn RDS	15	7 (4–11)	70	10 (8–12)	35	15 (10–20)	24	6 (3.6–8.3)	43	15 (11–19)	187	10 (7–15)
Jaundice	7	3 (1–6)	53	8 (6–10)	2	1 (0.1–3)	62	16 (12–19)	11	4 (2–6)	135	7 (3–17)

^a^Total sample size.

^b^95% CI adjusted for effect of clustering at hospital level.

^c^Number with data available per variable.

^d^Estimate.

^e^Birth weight: extremely low birth weight (LBW): <1 kg, very LBW: 1 to <1.5 kg, LBW: 1.5 to <2.5 kg, normal: 2.5 to <4 kg, macrosomia: ≥4 kg.

^f^These are disease episodes (only the top five are presented); a single patient may have more than one and thus counted in each separate diagnosis.

med, median; IQR, inter-quartile range; RDS, respiratory distress syndrome.

**Table 2.** Patterns of mortality in neonatal admissions

	H1 *n*^a^^ ^= 211		H2 *n*^a^^ ^= 693		H3 *n*^a^^ ^= 235		H4 *n*^a^^ ^= 400^b^		H5 *n*^a^^ ^= 297		Total *n*^a^^ ^= 1836	
Weight^e^	*n*^f^	%^c^(95% CI)	*n*^f^	%^c^(95% CI)	*n*^f^	%^c^(95% CI)	*n*^f^	%^c^(95% CI)	*n*^f^	%^c^(95% CI)	*n*^f^	%^d^(95% CI)
ELBW	2/3	67 (9–99)	13/18	72 (51–94)	2/2	100	2/2	100	13/13	100	32/38	84 (45–97)
VLBW	19/33	56 (39–73)	19/41	46 (31–62)	8/9	89	14/18	78 (58–98)	17/25	68 (49–87)	77/126	61 (42–77)
LBW	14/65	22 (11–32)	26/150	17 (11–23)	9/26	35 (16–53)	7/32	22 (7–37)	14/94	15 (8–22)	70/367	19 (14–25)
Normal BW	15/87	16 (9–24)	28/422	7 (4–9)	9/89	10 (4–16)	7/41	17 (5–29)	18/156	12 (7–17)	77/795	10 (8–12)
Macrosomia	3/8	38 (1–74)	3/31	9 (2–26)	0/11	–	0/2	–	1/6	17 (0.4–64)	7/58	10 (5–17)
Overall	53/196	26 (20–32)	89/662	13 (11–16)	28/137	22 (16–27)	30/95	29 (21–36)	63/294	21 (17–26)	263/1384	19 (17–22)

^a^Total sample size (number of cases).

^b^In H4 birth weight missing in 131 of 400(33%) and outcome missing in 256 of 400 (64%).

^c^Estimate.

^d^95% CI adjusted for effect of clustering at hospital level.

^e^Birth weight: extremely low birth weight (LBW): <1 kg, very LBW: 1 to <1.5 kg, LBW: 1.5 to <2.5 kg, normal: 2.5 to <4 kg, macrosomia: ≥4 kg.

^f^Number of deaths out of number of cases (case fatality).

A random effects logistic regression model with normal birth weight as the baseline category adjusted for sex and age at admission was fitted. The odds of death were significantly associated with extremely LBW [odds ratio (OR): 59, 95% CI: 21–158, *p* < 0.01], very LBW (OR: 14, 95% CI: 9–22, *p* < 0.01) and LBW (1500 to <2500 g; OR: 2.3, 95% CI: 1.6–3.3, *p* < 0.01) but similar for macrosomic babies (weight >4000 g, OR: 1.05, 95% CI: 0.5–2.8, *p* = 0.76).

## DISCUSSION

The MMR of 276 per 100 000 live births in these hospitals compares with a recent population level estimate for Kenya of 277.2 per 100 000 (95% CI: 175.4–414.1) in contrast with 12.1 of 100 000 (95% CI: 10.4–13.7) in developed countries [8]. A higher risk population is expected to deliver in hospitals, which may account for the recorded high proportion (20%) of teenage (13–19-year-old) mothers, who are known to have a higher risk of adverse neonatal and maternal outcomes [9–11]. Stillbirths have remained largely invisible; there were an estimated 2.65 million third-trimester stillbirths globally in 2008, 98% of which occurred in low- and middle-income countries [12, 13]. In the hospitals studied, there were 37 stillbirths per 1000 births with a rate of 20 per 1000 fresh stillbirths, for whom death is likely to have occurred intrapartum [14]. However, these fresh stillbirths may include early neonatal deaths, as misclassification between fresh stillbirths and early neonatal deaths is a challenge when enumerating still births [15].

The most common disease episodes were birth asphyxia (31%), prematurity/LBW (29%) and neonatal sepsis (14%) (Table 1). The number of patients with multiple diagnoses is not reported but previous work demonstrated considerable overlap in these three diagnoses [4]. In addition to many fresh stillbirths, the high numbers of birth asphyxia cases are of concern, contributing to a mortality of 10% in newborn units for normal weight admissions. Small babies remain a vulnerable population; a 2012 estimate suggested that 80% of neonatal deaths in Sub-Saharan Africa and South Asia were of this group [16]. We have shown that, even in hospitals where care should be available, 68% of neonatal deaths are <2500 g. Perhaps even more important for planning long-term service development, 41% of deaths were of birth weight <1500 g.

## CONCLUSION

The burden of maternal mortality, fresh stillbirths and birth asphyxia in these facilities suggests significant opportunity to earn the triple return on investment offered by improving referral and the quality of perinatal care [3]. In addition, given the disproportionately poor outcomes among LBW babies, enhancement of capacity to offer care for this vulnerable group is required.

## FUNDING

This work was supported by the Kenyan Ministry of Health, The Wellcome Trust, The Consortium for National Health Research (Kenya) and The University of Nairobi. JA s contribution was made possible by the Kenyan Ministry of Health and a grant from the Consortium for National Health Research (Kenya) to the SIRCLE Collaboration. ME has been supported by funds from The Wellcome Trust (076827, #097170). Additional funds from a Wellcome Trust Strategic Award (#084538) and a Wellcome Trust core grant awarded to the KEMRI-Wellcome Trust Research Programme (#092654) made this work possible. These grants supplemented salary support from the Ministry of Health (Kenya, to RN) and the University of Nairobi (to AW and FW). The Wellcome Trust and other funders had no role in developing this manuscript nor in the decision to submit for publication.
